# Rabies virus diversification in aerial and terrestrial mammals

**DOI:** 10.1590/1678-4685-GMB-2019-0370

**Published:** 2020-07-31

**Authors:** Rafael N. Oliveira, Caio C. Freire, Atila Iamarino, Paolo M. Zanotto, Rodrigo Pessoa, Sabri S. Sanabani, Sibele Pinheiro de Souza, Juliana G. Castilho, Helena B. C. R. Batista, Pedro Carnieli, Carla I. Macedo, Jaqueline T. Watanabe, Paulo E. Brandão

**Affiliations:** 1 Instituto Pasteur, Laboratório de Biologia Molecular, São Paulo, SP, Brazil.; 2 Universidade de São Paulo, Instituto de Ciências Biomédicas (ICB-II), Departamento de Microbiologia, Laboratório de Evolução Molecular e Bioinformática, São Paulo, SP, Brazil.; 3 Universidade de São Paulo, Instituto de Medicina Tropical de São Paulo, Departamento de Virologia, São Paulo, SP, Brazil.; 4 Dechra Brasil, Londrina, PR, Brazil.; 5 Universidade de São Paulo, Faculdade de Medicina Veterinária e Zootecnica, Departamento de Medicina Veterinária Preventiva e Saúde Animal, São Paulo, SP, Brazil.

**Keywords:** Rabies virus, genome, molecular evolution, phylogeny, heterotachy

## Abstract

Rabies is a fatal zoonotic infection of the central nervous system of mammals and has been known to humans for millennia. The etiological agent, is a neurotropic RNA virus in the order *Mononegavirales*, family *Rhabdoviridae*, genus *Lyssavirus*. There are currently accepted to be two cycles for rabies transmission: the urban cycle and the sylvatic cycle. The fact that both cycles originated from a common RABV or lyssavirus ancestor and the adaptive divergence that occurred since then as this ancestor virus adapted to a wide range of fitness landscapes represented by reservoir species in the orders *Carnivora* and *Chiroptera* led to the emergence of the diverse RABV lineages currently found in the sylvatic and urban cycles. Here we study full genome phylogenies and the time to the most recent common ancestor (TMRCA) of the RABVs in the sylvatic and urban cycles. Results show that there were differences between the nucleotide substitution rates per site per year for the same RABV genes maintained independently in the urban and sylvatic cycles. The results identify the most suitable gene for phylogenetic analysis, heterotachy among RABV genes and the TMRCA for the two cycles.

## Introduction

Rabies is endemic throughout the world apart from the Antarctic, Australia, Japan and New Zealand. It is caused by Rabies lyssavirus - RABV (order *Mononegavirales*; family *Rhabdoviridae*; genus *Lyssavirus*; specie *Rabies lyssavirus* [http://www.ictvonline.org/virusTaxonomy.asp]), a negative-sense ssRNA virus maintained in independent epidemiologic cycles by specific genera and species, mainly in the orders *Chiroptera* and *Carnivora* (Rupprecht *et al.*, 2002; Marston *et al.*, 2012; Mollentze *et al.*, 2014).

RABV is one of seventeen members of the genus *Lyssavirus* (King, A.M.K; Adans, M.J; Carstens, E.B; Lefkowitz 2012), fifteen of which have members of Chiroptera as exclusive reservoirs, showing the importance of this order as a reservoir for the genus (Rupprecht *et al.*, 2017; Hu *et al.*, 2018). This lends support to the hypothesis that various existing RABV lineages had an RABV specific to bats as a common ancestor (Badrane and Tordo, 2001; Hayman *et al.*, 2016; Velasco-Villa *et al.*, 2017) and that this ancestral lineage differentiated into the various lineages in the urban and sylvatic cycles (Oliveira *et al.*, 2010; Streicker *et al.*, 2010, 2012a,b; Kuzmin *et al.*, 2012). However, failure to isolate RABV or antibodies against this virus in bats in the Old World, particularly Asia, where the most ancient representatives of current RABV lineages are believed to be found, would appear to contradict this theory (Kuzmin and Rupprecht, 2007).

There are known to be two rabies transmission cycles: the urban cycle, in which the dog is the main reservoir and transmits the virus to other dogs, other domestic animals and man; and the sylvatic cycle, which is maintained by different terrestrial mammals and chiropterans (Acha and Szyfres, 2003).

Nevertheless, molecular evolution studies show that RABV differentiated into two major phylogenetic groupings (Badrane and Tordo, 2001; Bourhy *et al.*, 2008; Kuzmin *et al.*, 2012), which could be the basis for classification of rabies cycles into bat-related and dog-related cycles. The bat-related cycle (currently the sylvatic cycle) can be defined as all the RABV lineages maintained in different independent epidemiologic cycles exclusively in the Americas by wild animals, particularly chiropterans, while the dog-related cycle corresponds to all the lineages of rabies circulating in dogs (currently the urban cycle) and wild canids (currently the sylvatic cycle) maintained independently in multiple cycles around the world.

At least 35 RABV lineages have been described in the bat-related cycle, of which 31 are maintained in chiropterans. Of the four remaining lineages, two are maintained in skunks in Mexico and the south of the USA, one in racoons in the USA and one in the common marmoset (*C. jacchus*) in the northeast of Brazil. Eight major groups of genetic lineages are described in the dog-related cycle, for which the only reservoirs are members of the order Carnivora, particularly the domestic dog and wild canids (Bourhy *et al.*, 2008; Kuzmin *et al.*, 2012). Of these eight groups identified in the dog-related cycle, only the Africa 3 complex, for which the reservoir is the African yellow mongoose, is not maintained in dogs.

In view of the limited number of complete RABV genomes published, the uncertainty surrounding the classification of rabies transmission cycles and the lack of robust evolutionary analyses for RABV, our objective in this study was to generate data and understand the various divergence events for RABV isolated from terrestrial mammals and bats based on estimates of phylogeny, evolution rates and time to the most recent common ancestor (TMRCA) using complete genome sequences.

## Material and Methods

### RABV strains

Twenty-one RABV strains from different Brazilian rabies reservoirs (Table 1) isolated on the first passage in mouse brains, according to Koprowski (1996), were used for full genome sequencing. These isolates were obtained from vampire bats (*Desmodus* *rotundus*) and insectivorous bats (*Eptesicus furinalis*, *Myotis nigricans*, *Nyctinomops laticaudatus* and *Tadarida brasiliensis)*, crab-eating foxes (*Cerdocyon thous*), dogs, cattles and marmosets (*Callithrix jacchus)*, send to the virology laboratory of the Instituto Pasteur (São Paulo/Brazil) for rabies surveillance from 2006 to 2012.

**Table 1:** Temperature cycles used in PCR to amplify the RABV genome.

Cycle		Temperature	Time
1 X	{	94°C Denaturation	5 minutes
		94°C Denaturation	45 seconds
35 X		55°C Annealing	45 seconds
		68°C Extension	12 minutes
1 X		68°C Extension	10 minutes

These strains were classified in the RABV lineages *Myotis* I, *Eptesicus* I, *Eptesicus* II, *Nyctinomops*, *Tadarida brasiliensis* South America, *Desmodus rotundus*, *Cerdocyon thous* and *Callithrix jacchus* based on partial N or G gene sequences as previously described (Favoretto *et al.*, 2001; Carnieli *et al.*, 2008; Oliveira *et al.*, 2010; Almeida *et al.*, 2011).

This work complies with Protocol nº 2030/2010 issued by the “Ethics Committee in the use of animals” of the School of Veterinary Medicine and Animal Science of University of São Paulo.

### Full-genome RT-PCR

Total viral RNA was extracted from infected mouse brain with TRIzol ™ (Life Technologies, Carlsbad, CA, USA) according to the manufacturer’s instructions.

The double-stranded genome-length cDNA (genomic reverse transcription) was synthesized using the antisense primer “Final” (Campos *et al.*, 2011) targeting the 3’UTR 21 first nucleotides of trailer region of the antigenomic RNA (nucleotides 11904-11924 Rabies virus M13215.1 GenBank accession number) and RevertAid Premium Reverse Transcriptase (Fermentas ) (Oliveira, 2014).

For each sample, 15 μL of the extracted RNA was added to the mix for reverse transcription containing 8 μL 5X RT Buffer, 6 μL of the dNTP pool at 10 mM, 5 μL of the “Final” antisense primer at 10 μM , 400 U of Reverse Transcriptase enzyme RevertAid Premium Reverse Transcriptase (Fermentas ), 1 μL of RNAsin (Invitrogen ) and 35 μL of ultra-pure DNAse/RNAse free water to a final volume of 50 μL, carrying out reverse transcription at 42 ºC / 180 minutes followed by a 70 ºC / 15 minute reverse transcriptase inactivation cycle.

Genomic RABV amplicons (circa 12 kb) were obtained by PCR using a pair of primers targeting the leader and trailer regions of the RABV genome (Campos *et al.*, 2011), 1 μL of genomic cDNA and GoTaq™ Long PCR Master Mix 2X (Promega) according to the manufacturer’s instructions (Oliveira 2014).

The PCR protocol for the amplification of the complete RABV genome consisted of: 25 μL of GoTaq® Long PCR Master Mix 2X (Promega), 1 μL of the “Início” (sense) and “Final” (antisense) primers at a concentration of 10 μM, 1 μL of c-DNA and 22 μL of ultra-pure DNAse/RNAse free water to a final volume of 50 μL and taken to the thermocycler and subjected to the cycle described in Table 1.

### Whole viral genome library preparation and full-genome sequencing

Purified products were quantitated using Quant-IT HS reagents (Invitrogen, Life Technologies, Carlsbad, CA), and approximately 1 ng of each was used in a fragmentation reaction mix using a Nextera XT DNA sample prep kit according to the manufacturer’s protocol. Briefly, tagmentation and fragmentation of each product were simultaneously performed by incubation for 5 min at 55 °C followed by incubation in neutralizing tagment buffer for 5 min at room temperature. After neutralization of the fragmented DNA, a light 12-cycle PCR was performed with Illumina Ready Mix to add Illumina flowcell adaptors, indexes and common adapters for subsequent cluster generation and sequencing. Amplified DNA was then purified using Agencourt AMPure XP beads (Beckman Coulter), which excluded very short library fragments. Following AMPure purification, the quantity of each library was normalized to ensure equally library representation in our pooled samples. Prior to cluster generation, normalized libraries were further quantified by qPCR using the SYBR fast Illumina library quantification kit (KAPA Biosystems) following the instructions of the manufacturer. The qPCR was run on the 7500 Fast Real-Time PCR System (Applied Biosystems). The thermocycling conditions consisted of an initial denaturation step at 95 °C for 5 min followed by 35 cycles of [30 s at 95 °C and 45 s at 60 °C]. The final libraries were pooled at equimolar concentration and diluted to 4 nM. To denature the indexed DNA, 5 μL of the 4 nM library were mixed with 5 μL of 0.2 N fresh NaOH and incubated for 5 min at room temperature. 990 μL of chilled Illumina HT1 buffer was added to the denatured DNA and mixed to make a 20 pM library. After this step, 360 μL of the 20 pM library was multiplexed with 6 μL of 12.5 pM denatured PhiX control to increase sequence diversity and then mixed with 234 μL of chilled HT1 buffer to make a 12 pM sequenceable library. Finally, 600 μL of the prepared library was loaded on an Illumina MiSeq clamshell style cartridge for paired end 250 sequencing.

After they had been sequenced in an Illumina MiSeq^TM^, the reads were assembled without the use of reference genomes (*de novo* assembly) in CLC Genomics Workbench 6. When the assembled contigs in a genome were smaller than expected based on the size of the sequenced PCR fragment, they were extended by mapping the reads to the reference with 95% similarity and without gap opening or by using *de novo* assembly with the same similarity criterion in Geneious 6. Both the extended and the *de novo* assembled contigs had their reads mapped to the reference again and were evaluated in terms of their variability based on the number and quality of reads using the Quality-based Variant Detection function in CLC Genomics Workbench version 5.5.

Low-quality sequences were removed from the analysis, i.e., sequences with a phred Q score lower than 20, using at least 100 reads per site and applying a penalty for homopolymer regions (a quality reduction of 30% for every additional base). After editing, the regions of the RABV sequences corresponding to the *Início* and *Final* primers were removed.

### The dataset used for the analysis

The dataset used consisted of 138 complete RABV genome sequences from GenBank and the RABV genome sequences obtained in this study (Table 2). The sequences from GenBank were extracted with a PERL script using the criterion that they should have more than 10,000 nucleotides and that information about year, country and original host should be available. In all, there were 159 sequences, corresponding to 100 in the bat-related RABV cycle and 59 in the dog-related cycle. The region used for the analysis extended from nucleotide 59 to nucleotide 11801 (in relation to the PV strain, accession number M13215.1), *i.e.*, from the first nucleotide in the N gene messenger RNA to the stop codon in the L gene.

**Table 2:** Strains used in the study: accession number, reference, host, country and year of isolation.

GenBank accession number	Strain	*Host*	Country	Year
JQ685971.1	AZ3003	*Antrozous pallidus*	United States	2009
AB699220.1	BDR5	*Capra aegagrus hircus*	Bangladesh	2010
KM594025.1	IP5402/07	*Callithrix jacchus*	Brazil	2007
KM594024.1	IP1770/12	*Callithrix jacchus*	Brazil	2012
KM594023.1	IP 6634/08	*Callithrix jacchus*	Brazil	2008
JQ685973.1	A111043	*Canis latrans*	United States	2011
JQ685917.1	COCoyot2010	*Canis latrans*	United States	2010
KC196743.1	DRVNG11	*Canis lupus familiaris*	Nigeria	2011
HQ450385.1	DRVAH08	*Canis lupus familiaris*	China	2008
JN609295.1	FJDRV	*Canis lupus familiaris*	China	2008
FJ712194.1	D02	*Canis lupus familiaris*	China	2008
FJ712193.1	D01	*Canis lupus familiaris*	China	2008
KC660078.1	Fengtai	*Canis lupus familiaris*	China	2012
FJ866836.1	FJ009	*Canis lupus familiaris*	China	2008
FJ866835.1	FJ008	*Canis lupus familiaris*	China	2008
GU345746.1	CQ92	*Canis lupus familiaris*	China	1992
GU358653.1	GX4	*Canis lupus familiaris*	China	1994
JQ730682.1	CYN1009D	*Canis lupus familiaris*	China	2010
EU293113.1	9001FRA	*Canis lupus familiaris*	French Guiana	1990
KF154999.1	RV2417	*Canis lupus familiaris*	United Kingdom	2008
KF154998.1	RV2324	*Canis lupus familiaris*	Israel	1950
KM594039.1	IP 7841/09	*Canis lupus familiaris*	Brazil	2009
AB517659.1	BRdg335	*Canis lupus familiaris*	Brazil	2003
KF155002.1	RV2772	*Canis lupus familiaris*	Tanzania	2010
JQ944706.1	1352KRA	*Canis lupus familiaris*	Russia	2008
JQ944705.1	1350KRA	*Canis lupus familiaris*	Russia	2008
AB206409.2	BR_Pfx3	*Cerdocyon thous*	Brazil	2001
AB362483.1	BR_Pfx1	*Cerdocyon thous*	Brazil	2002
KC169986.1	GXHXN	*Bos taurus*	China	2009
KC193267.1	CNM1101C	*Bos taurus*	China	2011
JQ685936.1	3634DR	*Bos taurus*	Mexico	2009
KM594043.1	IP 4005/12	*Bos taurus*	Brazil	2012
KF155001.1	RV2627	*Bos taurus*	Morocco	2009
KF155000.1	RV2516	*Bos taurus*	Iraq	2010
KC171643.1	08F40	*Bos taurus*	South Korea	2008
KM594041.1	IP 2990/13	*Desmodus rotundus*	Brazil	2013
KM594042.1	IP 2991/13	*Desmodus rotundus*	Brazil	2013
KM594040.1	IP 2992/13	*Desmodus rotundus*	Brazil	2013
JQ944707.1	1410KOM	*Cervidae*	Russia	2008
JQ685942.1	AZBAT65094	*Eptesicus fuscus*	United States	1981
JQ685931.1	WA0173	*Eptesicus fuscus*	United States	2000
JQ685903.1	CA04148	*Eptesicus fuscus*	United States	2004
JQ685961.1	AZ10140	*Eptesicus fuscus*	United States	2010
JQ685950.1	A093504	*Eptesicus fuscus*	United States	2009
JQ685897.1	SM5442	*Eptesicus fuscus*	United States	2001
JQ685923.1	SM4871	*Eptesicus fuscus*	United States	1999
JQ685926.1	AZ2408	*Eptesicus fuscus*	United States	2005
JQ685960.1	SM4872	*Eptesicus fuscus*	United States	2001
JQ685956.1	AZBAT7453	*Eptesicus fuscus*	United States	1975
JQ685909.1	CA100	*Eptesicus fuscus*	United States	2005
JQ685907.1	SM3849	*Eptesicus fuscus*	United States	1996
JQ685913.1	AZBAT6763	*Eptesicus fuscus*	United States	1985
JQ685974.1	SM3844	*Eptesicus fuscus*	United States	1995
JQ685898.1	A093500	*Eptesicus fuscus*	United States	2009
JQ685946.1	SM4862	*Eptesicus fuscus*	United States	1999
JQ685951.1	AZ10144	*Eptesicus fuscus*	United States	2010
JQ685920.1	EF	*Eptesicus fuscus*	United States	1984
JQ685925.1	WAEF03	*Eptesicus fuscus*	United States	2004
KM594028.1	IP 346/10	*Eptesicus furinalis*	Brazil	2010
KM594027.1	IP 230/10	*Eptesicus furinalis*	Brazil	2010
KM594026.1	IP 512/09	*Eptesicus furinalis*	Brazil	2009
KM594029.1	IP 3208/06	*Eptesicus furinalis*	Brazil	2006
JQ647510.1	WH11	*Equidae*	China	2011
JQ423952.1	BJ2011E	*Equidae*	China	2011
JQ685945.1	SM6709	*Felis silvestris catus*	United States	2005
KC595281.1	RusLipetsk8053c	*Felis silvestris catus*	Russia	2011
JQ685948.1	OR05455	*Urocyon cinereoargenteus*	United States	2010
JQ685977.1	OR58	*Urocyon cinereoargenteus*	United States	2010
JQ685924.1	OR704	*Urocyon cinereoargenteus*	United States	2010
JQ685914.1	OR703	*Urocyon cinereoargenteus*	United States	2010
JQ685933.1	SM5950	*Urocyon cinereoargenteus*	United States	2004
JQ685934.1	2401	*Urocyon cinereoargenteus*	United States	2009
JQ685972.1	2395	*Urocyon cinereoargenteus*	United States	2009
JQ685939.1	2399	*Urocyon cinereoargenteus*	United States	2009
JQ685892.1	2398	*Urocyon cinereoargenteus*	United States	2009
JQ685908.1	1060	*Urocyon cinereoargenteus*	United States	2009
JQ685928.1	2403	*Urocyon cinereoargenteus*	United States	2009
JQ685896.1	2402	*Urocyon cinereoargenteus*	United States	2009
JQ685912.1	2400	*Urocyon cinereoargenteus*	United States	2009
JQ685957.1	OR8767	*Urocyon cinereoargenteus*	United States	2009
JQ685918.1	OR05506	*Urocyon cinereoargenteus*	United States	2010
EU293115.1	9147FRA	*Vulpes sp.*	France	1991
JQ944708.1	1564NNO	*Vulpes vulpes*	Russia	2008
KC595280.1	RusLipetsk8052f	*Vulpes vulpes*	Russia	2011
KC595283.1	RusLipetsk8057f	*Vulpes vulpes*	Russia	2011
KC595282.1	RusLipetsk8054f	*Vulpes vulpes*	Russia	2011
JQ685899.1	A100515	*Urocyon cinereoargenteus*	United States	2009
JQ685943.1	A100511	*Urocyon cinereoargenteus*	United States	2009
GU345747.1	J	*Homo sapiens*	China	1986
EU643590.1	HN10	*Homo sapiens*	China	2006
EU293111.1	8764THA	*Homo sapiens*	Thailand	1983
JQ685953.1	3645DR	*Homo sapiens*	Mexico	2009
AB569299.1	H081320	*Homo sapiens*	Sri Lanka	2008
KC737850.1	A115300	*Homo sapiens*	United States	2011
KF154996.1	RV61	*Homo sapiens*	United Kingdom	1987
JX473839.1	192J09	*Canis mesomelas*	Namibia	2009
JX473838.1	178J09	*Canis mesomelas*	Namibia	2009
JQ685916.1	FL1010	*Lasiurus intermedius*	United States	2002
JQ685915.1	TX4904	*Lasiurus intermedius*	United States	2002
JQ685895.1	WA1185	*Lasionycteris noctivagans*	United States	2003
JQ685902.1	TN209	*Lasiurus borealis*	United States	2005
JQ685910.1	TX5960	*Lasiurus xanthinus*	United States	2002
JQ685900.1	FL769	*Lasiurus seminolus*	United States	2003
JQ685919.1	NJ2262	*Lasiurus borealis*	United States	2005
JQ685947.1	TN310	*Lasiurus cinereus*	United States	2004
JQ685921.1	FL1078	*Myotis austroriparius*	United States	2001
JQ685927.1	SM5080	*Mephitis mephitis*	United States	2001
JQ685893.1	SM5079	*Mephitis mephitis*	United States	2001
JQ685932.1	SM5076	*Mephitis mephitis*	United States	2001
JQ685949.1	SM5075	*Mephitis mephitis*	United States	2001
JQ685966.1	SM5470	*Mephitis mephitis*	United States	2001
JQ685935.1	SM5074	*Mephitis mephitis*	United States	2001
JQ685906.1	SM5102	*Mephitis mephitis*	United States	2001
JQ685958.1	SM5101	*Mephitis mephitis*	United States	2001
JQ685904.1	SM5081	*Mephitis mephitis*	United States	2001
JQ685962.1	SM5441	*Mephitis mephitis*	United States	2001
JQ685969.1	SM5440	*Mephitis mephitis*	United States	2001
JQ685959.1	SM5451	*Mephitis mephitis*	United States	2001
JQ685940.1	SM5100	*Mephitis mephitis*	United States	2001
JQ685911.1	SM5077	*Mephitis mephitis*	United States	2001
JQ685930.1	SM5103	*Mephitis mephitis*	United States	2001
JQ685964.1	SM5596	*Mephitis mephitis*	United States	2004
JQ685941.1	SM1545	*Mephitis mephitis*	United States	2005
JQ685968.1	A100512	*Mephitis mephitis*	United States	2009
JQ685938.1	A100514	*Mephitis mephitis*	United States	2009
JQ685894.1	CA982	*Mephitis mephitis*	United States	1994
JQ685970.1	CASK2	*Mephitis mephitis*	United States	1974
JQ685944.1	NC839	*Mephitis mephitis*	United States	1984
KC762941.1	JX0917fb	*Melogale moschata*	China	2009
GU647092.1	JX0845	*Melogale moschata*	China	2008
FJ712195.1	F02	*Melogale moschata*	China	2008
FJ712196.1	F04	*Melogale moschata*	China	2008
JQ685955.1	AZ4490	*Myotis yumanensis*	United States	2005
KM594032.1	IP 497/09	*Myotis nigricans*	Brazil	2009
KM594031.1	IP 163/10	*Myotis nigricans*	Brazil	2010
KM594030.1	IP 1400/10	*Myotis nigricans*	Brazil	2010
KM594034.1	IP 350/10	*Nyctinomops laticaudatus*	Brazil	2010
KM594036.1	IP 542/10	*Nyctinomops laticaudatus*	Brazil	2010
KM594035.1	IP 412/10	*Nyctinomops laticaudatus*	Brazil	2010
JQ685963.1	Coati3639	*Nasua narica*	Mexico	2009
JQ944704.1	184VNO	*Nyctereutes procyonoides*	Russia	2009
KC171645.1	BV9901PJ	*Nyctereutes procyonoides*	South Korea	1999
KC171644.1	BD0406CC	*Nyctereutes procyonoides*	South Korea	2004
JQ685952.1	A022971	*Parastrellus hesperus*	United States	2002
JQ685965.1	A022972	*Parastrellus hesperus*	United States	2002
JQ685901.1	RAC	*Procyon lotor*	United States	2003
EU311738.1	RRVON992	*Procyon lotor*	Canada	1999
JQ685922.1	TN186	*Perimyotis subflavus*	United States	2005
AB635373.1	H141309	*Paradoxurus zeylonensis*	Sri Lanka	2009
JQ685954.1	MEXSK13938	*Spilogale putorius*	Mexico	2007
JQ685929.1	MEXSK3644	*Spilogale putorius*	Mexico	2009
JQ685975.1	MEXSK3636	*Spilogale putorius*	Mexico	2009
EU293116.1	9704ARG	*Tadarida brasiliensis*	Argentina	1997
KM594038.1	IP 1586/10	*Tadarida brasiliensis*	Brazil	2010
KM594037.1	IP 3176/09	*Tadarida brasiliensis*	Brazil	2009
KM594033.1	IP 4431/10	*Tadarida brasiliensis*	Brazil	2010
JQ685905.1	FL385	*Tadarida brasiliensis*	United States	2003
JX473841.1	240K09	*Tragelaphus strepsiceros*	Namibia	2009
JX473840.1	239K09	*Tragelaphus strepsiceros*	Namibia	2009

The only sequences used were those that did not show any evidence of recombination events when examined by the RDP, GenConv, Chimaera, MaxChi, Bootscan, SiScan and 3Seq methods implemented in RDP4 (beta 4.8 version) (Martin *et al.*, 2010) with a 95% confidence interval and Bonferroni correction for multiple comparisons.

### Phylogenetic signal analysis

The phylogenetic signal content for the RABV genome and each RABV gene was investigated with the likelihood mapping algorithm (Strimmer and von Haeseler, 1997) implemented in TREE-PUZZLE v 5.2 (Schmidt *et al.*, 2002), which quantifies the well-resolved phylogenetic quartets in the database.

### Heterotachy analysis

Genome sequences were used in the heterotachy analysis with PAML v 4.6 (Yang, 2007) to test the following hypotheses: *H*_*0*_ - the viral lineages in both RABV cycles are under the same molecular clock, and *H*_*A*_ - the lineages in each cycle are under different clocks.

One hundred phylogenetic trees were constructed using the ML (maximum likelihood) method with GARLI v 2.0 (Bazinet *et al.*, 2014), and the tree with the highest value ML was used.

To test the significance of the difference between the likelihood (L) values for each model studied, the likelihood ratio test (LRT) was used according to the equation next.

LRT = 2 x (L1 - L2) where L1 is equal to the likelihood of model 1 and L2 the likelihood of model 2. In this case, the degrees of freedom employed were equal to 157, which is equivalent to the number of taxa -2 (N-2). The level of significance established was 0.05, which critical value is 206.390, in a chi-square table.

When the hypothesis of different molecular clocks for the RABV genomes in the two cycles was confirmed, analyses were carried out for each cycle and for each gene to determine the genes or regions of the RABV genome responsible for this phenomenon so that they could then be removed from the joint analyses of the cycles.

Estimates of the nucleotide substitution rate per site per year, estimates of the TMRCA and phylogeny estimates

Estimates of the nucleotide substitution rates per site per year and the time to the most recent common ancestor (TMRCA) for genome sequences in which heterotachy was detected were initially made for each of the five genes for each cycle separately. After this analysis, only those genes with similar substitution rates in both cycles were used to estimate the TMRCA and carry out the phylogenetic analysis.

Substitution rates per site per year were calculated in BEAST v 1.7.4 (Drummond *et al.*, 2012). Before the analysis in BEAST, initial substitution rates (μ) for use as priors in the Bayesian analyses were estimated with Parth-O-Gen (http://tree.bio.ed.ac.uk/software/tempest/).

Maximum clade credibility (MCC) trees were inferred for each of the five RABV genes using a Monte Carlo Markov Chain (MCMC) approach implemented in BEAST v 1.7.4 (Drummond *et al.*, 2012) and because RABV are under purifying selection (Troupin *et al.*, 2016) we employed the SRD6 model (Shapiro *et al.*, 2006) for coding sequences to avoid underestimation of TMRCA (Wertheim and Pond, 2011) with an uncorrelated relaxed molecular clock and a lognormal distribution. The previously estimated nucleotide substitution rate (μ) was used in the model.

The MCMC converged after two independent runs with 50 million generations each. Sampling was performed for every 5000 trees, which was sufficient to obtain a sample of the stationary MCMC. This was examined in Tracer v 1.5 (http://tree.bio.ed.ac.uk/software/). An effective sample size (ESS) of greater than 200 was considered sufficient.

Estimates of nucleotide substitution rates per site per year, estimates of TMRCA for the bat-related and dog-related RABV cycles and phylogeny estimates using the concatenated G-L genes

MCC trees based on the concatenated G and L genes were inferred using an MCMC approach implemented in BEAST v 1.7.4 (Drummond *et al.*, 2012) and the SRD6 model with an uncorrelated relaxed molecular clock and a lognormal distribution (Drummond *et al.*, 2006a). The previously estimated nucleotide substitution rate (μ) was used in the model. The MCMC converged after two independent runs with 50 million generations each. Sampling was performed for every 5000 trees. This was sufficient to obtain a sample of the stationary MCMC, which was examined in Tracer v 1.5 (http://tree.bio.ed.ac.uk/software/). An effective sample size (ESS) of greater than 200 was considered sufficient.

## Results

### Full-genome sequencing

The 21 DNA samples from amplified RABV genomes were sequenced successfully; after assembly and editing they yielded DNA sequences corresponding to nucleotide 23 to nucleotide 11911 in the fixed PV strain (M13215.1), or the complete genomes without the primer hybridization regions. The sequences were deposited in GenBank under accession numbers KM594023 - KM594043.

### Phylogenetic signal analysis

The results of the phylogenetic signal analysis showed that the RABV G gene is the most suitable for use in phylogenetic studies involving the bat-related and dog-related RABV cycles as it provided a better resolution than any of the four other genes or the complete genome (Table 3).

**Table 3:** Description of the phylogenetic signal found for each RABV gene and for the complete genome using the quartet method.

Genomic region	% Resolved quartets	% Unresolved quartets	% Partially resolved quartets
N protein	95.9	1.7	2.4
P protein	94.9	2.5	2.6
M protein	94.0	2.7	3.3
G protein	99.6	0.1	0.3
L protein	98.9	0.3	0.8
Genome	99.3	0.1	0.6

### Heterotachy analysis

The hypothesis that the viral lineages in both RABV cycles are under the same molecular clock was less favored than the hypothesis that the lineages in each cycle are under different clocks. This finding was inferred after observing a lower likelihood value for the first model, with a significant difference between the values obtained for each model (LRT = 2964.948), considering the critical (206.39) value in a chi-square table.

L1 =-246446.323 L2 = -244963.849 LTR = 2964.

Heterotachy was observed in RABV from bat-related and dog-related cycles. Subsequent analyses were therefore carried out separately gene by gene for each cycle in order to identify the genes responsible for this phenomenon so that the phylogenetic analyses for the bat-related and dog-related RABV cycles could be carried out together using the most suitable gene(s) for this purpose.

Estimates of the nucleotide substitution rate per site per year, estimates of the TMRCA and phylogeny estimates

This analysis was first carried out separately for all the five RABV genes for each cycle. The results are shown in Table 4.

**Table 4:** Nucleotide substitution rates per site and TMRCA for the N, P, M, G and L genes separately in the aerial and terrestrial cycles and for both cycles together using the G and L genes concatenated.

Gene	Substitution rate		TMRCA	
	Aerial	Terrestrial	Aerial	Terrestrial
Protein N	1.30E-4 (1.22E-4 - 1.38 E-4)	2.26E-4 (1.40 E-4 - 3.14E-4)	998.524 (754.16 - 1266.86)	594.90 (370.228 - 876.97)
Protein P	2.28 E-4 (2.04E-4 - 2.50E-4)	1.17E-4 (6.9E-5 - 1.64E-4)	710.11 (517.1 - 935.39)	1374.14 (839.82 - 2067.74)
Protein M	2.49 E-4 (2.37E-4 -2.60E-4)	1.17 E-4 (6.79 E-5 - 1.62 E-4)	608.61 (450.84 - 780.42)	1100.99 (639.20 - 1679-46)
Protein G	1.4E-4 (1.33E-4 - 1.47E-4)	1.18 E-4 (7.19E-5 - 1.65E-4)	1099.35 (807.90 - 1419.75)	1264.93 (771.61 - 1844.29)
Protein L	1.15E-4 (1.13E-4 - 1.16E-4)	1.34E-4 (1.32E-4 - 1.36E-4)	1209.79 (974.65 - 1464.61)	1254.59 (947.11 - 1622.08)
G+L	1.14E-4 (7.09E-5 - 1.54 E-4)		1872.16 (1178 - 2723)	

In the dog-related cycle, the highest substitution rates were found for the N gene (2.26 E-4). These were approximately twice the rates for the other genes, all of which appear to be evolving at approximately similar rates with overlapping confidence intervals: P (1.17 E-4), M (1.17 E-4), G (1.18 E-4) and L (1.34 E-4). This result suggests that the constraints on nucleotide substitutions per site in the RABV N gene in the dog-related cycle are smaller than those for the P, M, G and L genes.

In the bat-related cycle the mean substitution rates for the P and M genes (2.28 E-4 and 2.49 E-4, respectively) were similar to each other but different from those for the N, G and L genes (1.30 E-4, 1.4 E-4 and 1.15 E-4, respectively), which were also similar to each other. Unlike in the dog-related cycle, in the bat-related cycle the genes with the smallest constraints on nucleotide substitutions would appear to be the P and M genes.

The results for intercycle heterotachy showed that the N gene appears to be accumulating nucleotide substitutions in the dog-related cycle twice as fast as in the bat-related cycle (2.26 E-4 and 1.30 E-4, respectively) with non-overlap of confidence intervals. This finding indicates that the constraints on nucleotide substitutions per site in the N gene in RABV circulating in the bat-related cycle are greater than those in the same gene in the dog-related cycle.

The P and M genes in the bat-related cycle had higher substitution rates than in the dog-related cycle with non-overlap of confidence intervals, indicating that the constraints on nucleotide substitutions in these genes may be greater in the dog-related cycle than in the bat-related cycle.

The L gene exhibited weaker heterotachy, and the higher rates were in the dog-related cycle (1.32 E-4 - 1.36 E-4 compared with 1.13 E-4 - 1.16 E-4 in the bat-related cycle).

Only the G gene did not exhibit any heterotachy between the cycles, suggesting that nucleotide substitution in both cycles may be subject to the same constraints.

To infer the divergence time between the lineages in the bat-related and dog-related cycles, the analysis was repeated but this time using both cycles together and only the G and L genes concatenated as these not only are evolving at approximately similar rates in both cycles (Table 3) but also provide the best phylogenetic signal for RABV (Table 2).

**Figure 1:**
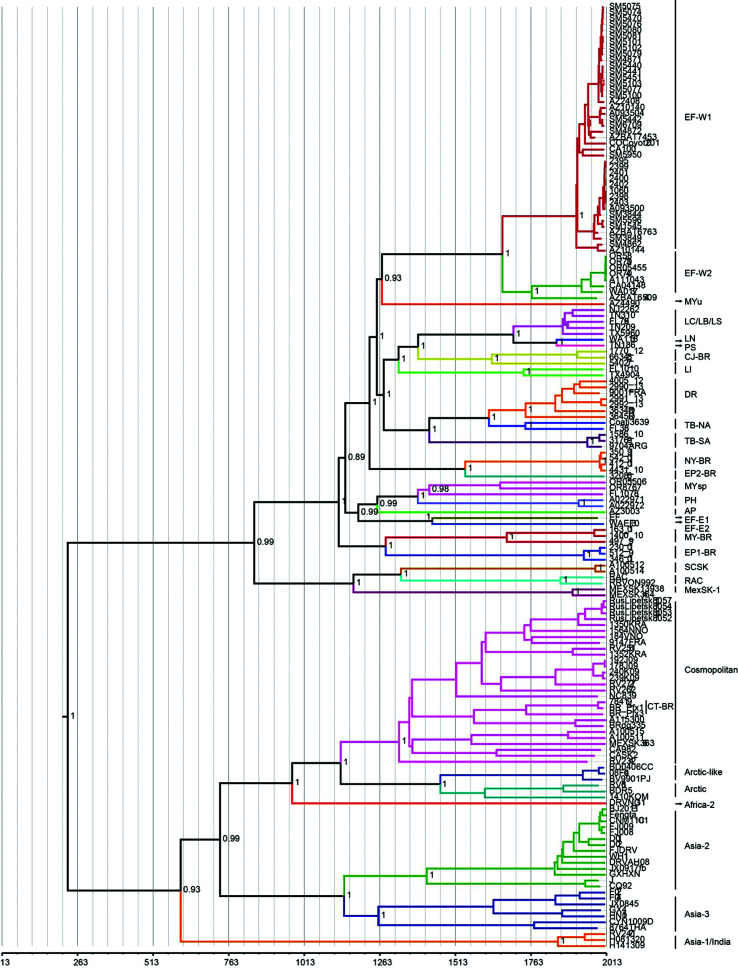
Maximum clade credibility tree inferred from the concatenated G and L genes. The branches are calibrated according to the year when the lineage was isolated. The scale at the bottom of the graph is in years and can be used to identify when the separation events between RABV lineages occurred. The groups are color-coded according to the classification proposed by Bourhy *et al.* (2008), and Kuzmin *et al.* (2012).

The Bayesian inference tree built with the concatenated G and L genes (Figure 1) indicated that RABV separated into bat-related and dog-related cycles around the year 230 CE. Most of the RABV lineages found in these two cycles according to the classification proposed by Bourhy *et al.* (2008) and Kuzmin *et al.* (2012), are shown in this figure. Also shown in the figure the Brazilian RABV lineages sequenced in this study: *Myotis* Brasil (MY-BR), *Eptesicus* 1 Brasil (EP1-BR), *Eptesicus* 2 Brasil (EP2-BR), *D. rotundus* (DR), *Nyctinomops* Brasil (NY-BR), *T. brasiliensis* South America (TB-SA*), C. jacchus* (CJ-BR) and *C. thous* (CT-BR).

## Discussion

Although sequencing of complete genomes from different RABV isolates has been performed since 1986 (Tordo *et al.*, 1986), to our knowledge DNA sequencing of complete RABV genomes using a single RT-PCR has not been described to date.

If only one amplification step is carried out to obtain the whole RABV genome sequence, the resulting sequence is more reliable as the use of different amplicons can, even if these are from the same viral sample, generate amplicons of different viral subpopulations for each of the segments being studied (Drummond *et al.*, 2006b; Lauring *et al.*, 2012).

As previously mentioned, in this study we investigated 25 of the 35 lineages already described for the bat-related RABV in the Americas (Favoretto *et al.*, 2001; Drummond *et al.*, 2006b; Oliveira *et al.*, 2010; Streicker *et al.*, 2010; Almeida *et al.*, 2011; Kuzmin *et al.*, 2012; Lauring *et al.*, 2012), of which three (LC, LB and LS) grouped into a single monophyletic cluster. Of the eight Brazilian lineages for which virtually the complete genomes were sequenced here, the lineages *Nyctinomops* (NY-BR), *Eptesicus* Brazil1 (EP-BR1), *Eptesicus* Brazil 2 (EP-BR2), *Myotis* Brazil (MY-BR) and *C*. *jacchus* (CJ-BR) had all their genes sequenced for the first time (Figure 1).

According to the nucleotide substitution rates found for the five RABV genes in the bat-related and dog-related cycles (Table 4), we can conclude that only the G and L genes are accumulating nucleotide substitutions at similar rates per site per year in both cycles and that these genes provide the best phylogenetic signal of all five RABV genes (Table 3). Hence, phylogenetic analyses that use nucleotide substitution rates per site per year to establish the TMRCA for RABV should not use the complete genome but only the genes that appear to be evolving at similar rates in the various lineages in the dog-related and bat-related cycles and that provide the best phylogenetic signal.

The results for the TMRCA for the bat-related and dog-related RABV cycles inferred from the concatenated G and L genes indicate that the divergence occurred around 140 AD (Table 4). In the MCC phylogenetic tree the median value of the node corresponding to the divergence between the two cycles places this divergence in the year 230 AD (Figure 1). Differences of this nature are inherent to Bayesian inferences (Drummond *et al.*, 2005, 2012).

Using 151 sequences from the complete N gene (25 sequences from the bat-related cycle and 126 from the dog-related cycle) Bourhy *et al.* (2008) inferred that the two cycles separated around 1250 AD. In the same study, when they used 74 sequences from the complete G gene (2 from the bat-related cycle and 72 from the dog-related cycle), authors dated this divergence to 1400 AD.

Nevertheless, our results show that although it is the most conserved of the RABV genes, the N gene neither provides the best phylogenetic signal nor has similar nucleotide substitution rates per site per year in both cycles and is therefore not the ideal gene for estimating the TMRCA. This, together with the small number of bat-related lineages and sequences studied by Bourhy *et al.* (2008) in the bat-related cycle, particularly in the case of the G gene, is the likely cause of the differences in the results for the TMRCA in the two cycles between this study and the study by Bourhy *et al.*, 2008.

Based on the MCC tree built using concatenated G and L genes, the median node dates corresponding to the origin of each cycle are approximately 850 AD for the bat-related cycle (a posterior of 0.99) and 600 AD for the dog-related cycle (a posterior of 0.93). This would indicate that the representatives of RABV in the dog-related cycle are older and have been evolving for longer than those of RABV in the bat-related cycle, although the confidence intervals overlap, which is expected as the lineages in each cycle diverged at about the same time. One of the hypotheses for this difference in the data of origin of each cycle is the representativeness of the RABV sequences used in the analysis, as these may not include the oldest lineages in each cycle.

All the existing RABV lineages maintained in the dog-related cycle shared a common ancestor with the Asia 1/India domestic-dog lineage around 600 AD. Asia 1/India occupies the most basal position in this group, suggesting that this lineage, which circulates in southern India, was the first to diverge and thus the oldest circulating in the dog-related cycle. This would corroborate the findings reported by Bourhy *et al.* (2008).

Troupin *et al.* (2016), using the five concatenated RABV genes and dog-related lineages, estimated this divergence to have occurred between the years 1308 and 1510 AD. This variation lies within the confidence intervals of the estimates calculated here using the concatenated bat-related and dog-related RABV G and L genes as well as each of the five RABV genes when the TMRCA using only dog-related RABV was assessed (Table 4). The discrepancy regarding the mean values is probably because Troupin *et al.* (2016) did not include bat-related RABV strains in their analysis and therefore did not take into account the common origin of bat- and dog-related RABV.

Similarly, Velasco-Villa *et al.* (2017), using the N gene, estimated that dog-related RABV diverged between 1273 and 1562 AD, a result also found in the present study using all genes separately and lineages from the dog-related cycle (Table 4), as bat-related RABV lineages were not used in the study by Velasco-Villa *et al.* (2017).

The Cosmopolitan complex of lineages, which includes those maintained in canids in Brazil, was estimated to have originated around 1300 AD. The basal group in this complex is a sample isolated in the 1950s in a dog in Israel (RV2324 – KF154998.1). In their study of the phylogeography of canine rabies around the world, Bourhy *et al.* (2008) found that the basal group for the Cosmopolitan complex was a sample isolated in a human in the 1970s in Egypt (8692EGY - U22627), which is genetically very similar to the RV2324 isolate.

The isolate from Egypt shares an ancestor with the other lineages in the Cosmopolitan complex, which includes the lineage maintained by wild canids in South America, lineages maintained by the striped skunk and wild canids in North America and some lineages maintained by wild canids in Eurasia and dogs in Africa *(* Kuzmin *et al.*, 2012).

The lineages maintained in Brazil in independent epidemiologic cycles by the crab-eating fox (*Cerdocyon thous*) (CT-BR lineage) and domestic dog (Domestic dog-BR) apparently shared a common ancestor and have evolved independently in each of these reservoirs since approximately 1570 AD, corroborating the hypothesis that rabies in wild canids in the Americas is associated with European colonization of the continent from the 14^th^ century onwards (Baer, 2007).

As already shown by other authors (Bourhy *et al.*, 2008; Kuzmin *et al.*, 2012), in the MCC phylogenetic tree inferred from the concatenated G and L genes (Figure 1), the MexSK-1, SCSK and RAC lineages, which are maintained in the eastern spotted skunk (*Spilogale putorius*), the striped skunk (*Mephitis mephitis*) and the raccoon (*Procyon lotor*), respectively, share a common ancestor with all the other lineages in the bat-related cycle maintained by bats and the common marmoset (*C. jacchus*). The divergence between these two groups occurred around 850 AD. Although the reservoir of this ancestral RABV remains unknown, some authors have put forward the hypothesis that this original reservoir of the rabies virus in the Americas was a chiropteran (Badrane and Tordo, 2001; Hayman *et al.*, 2016; Velasco-Villa *et al.*, 2017).

In the bat-related cycle, the common ancestor of the complex of lineages maintained in North American carnivores and its diversification into the current lineages was dated in the present study to approximately 1170 AD, while the common ancestor of RABV maintained in chiropterans and its diversification into the many existing lineages was inferred to around 1110 AD. For both estimates, the confidence intervals overlapped.

Among the lineages in the bat-related cycle maintained by bats and *C*. *jacchus*, the most basal group consists of two Brazilian lineages, *Myotis* Brazil (MY-BR) and *Eptesicus* Brazil 1 (EP1-BR), which shared a common ancestor in around 1280 AD (Figure 1). However, this result should be interpreted with caution, as this basal position was not confirmed in ML analyses performed with the complete genome (Kotait *et al.*, 2019), and many lineages in this cycle were not included in the present study.

The Brazilian lineage *C. jacchus* (CJ-BR), the only RABV lineage which has a primate as its reservoir (Kotait *et al.*, 2019), was estimated here to have originated around 1600 AD. It shared an ancestor around 1400 AD with RABV lineages maintained by different species of bats in the genus *Lasiurus* (LB, LC, LS) and the species *Lasionycteris noctivagans* (LN) and *Perimyotis subflavus* (PS) in North America (Figure 1). The fact that the LC lineage also circulates in bats in the genus *Lasiurus* in Brazil (Oliveira *et al.*, 2010) makes this common origin quite plausible.

Although the lineages *Nyctinomops* Brazil (NY-BR) and *Eptesicus* Brazil 2 (EP2-BR) have already been described by other authors (Kobayashi *et al.*, 2005, 2007a,b; Baer, 2007; Oliveira *et al.*, 2010; Albas *et al.*, 2011; Almeida *et al.*, 2011), their probable divergence from a common ancestor around 1550 AD has not been reported in the literature to date.

The close relationship between the lineages *T. brasiliensis* North America (TB-NA) and *D. rotundus* (DR) is already known, and our findings suggest that the divergence between the two lineages occurred around 1600 AD. As suggested in other studies, the *T. brasiliensis* South America (TB-SA) lineage is probably the basal group for the *T. brasiliensis* North America and *D. rotundus* lineages (Kobayashi *et al.*, 2005; Oliveira *et al.*, 2010; Kuzmin *et al.*, 2012), and the divergence from the common ancestor that gave rise to the TB-NA and DR lineages probably occurred around 1400 AD.

While the geographic distribution of the RABV lineages can be clearly seen in the dog-related cycle (showing the migration from India to Eurasia and Africa and finally the Americas as a result of the great maritime expeditions in the 15^th^ century), the geographic isolation is not so readily apparent in the lineages in the bat-related cycle. The clustering of the lineages in this cycle is primarily a result of the specific nature of the reservoirs, although certain populations in some lineages in this cycle have a geographic distribution (Streicker *et al.*, 2010, 2012a,b). Nevertheless, *L. cinereus* has already been isolated in bats of the genus *Lasiurus* in North America and Brazil, showing that this lineage circulates between continents (Oliveira *et al.*, 2010; Streicker *et al.*, 2010; Kuzmin *et al.*, 2012; Svitek *et al.*, 2014).

Given the representativeness of the data used in this study, we can consider there to be five host genera (*Canis*, *Vulpes*, *Nyctereutes*, *Mephitis* and *Cynictis*) acting in the selective processes involved in the adaptation and selection of representative lineages in the dog-related cycle and at least thirteen (*Mephitis*, *Procyon*, *Callithrix*, *Myotis*, *Eptesicus*, *Lasiurus*, *Nyctinomops*, *Tadarida*, *Perimyotis*, *Lasionycteris*, *Desmodus*, *Parastrellus* and *Antrozous*) acting in the bat-related cycle. In the latter cycle, species-specific lineages can be found in certain genera (Favoretto *et al.*, 2001; Oliveira *et al.*, 2010; Streicker *et al.*, 2010; Kuzmin *et al.*, 2012).

Like the vast majority of RNA viruses, the RABV RNA-dependent RNA polymerase complex formed by the P and L proteins does not have any proofreading 3’ to 5’ exonuclease activity. This, together with the large number of progeny and the short interval between RABV life cycles, leads to a high mutation rate with a high probability of neutral mutations getting fixed in mutant subpopulations that maintain themselves by replicating at a lower frequency than the original (master) population in their reservoirs. When this complex composite viral population faced sudden habitat change as a result of, for example, interspecific transmission events, these heterogeneous viral populations may have been responsible for improved adaptability in a new host, leading to intraspecific transmission and the emergence of new RABV lineages or a newly dominant consensus (Streicker *et al.*, 2010, 2012a; Lauring *et al.*, 2012; Borucki *et al.*, 2013).

Different evolutionary patterns resulting from adaptation and evolution in different reservoirs can be reflected in a variety of nucleotide substitution rates in a given site and year for the same gene in RABV distributed between the bat-related and dog-related RABV. Such evolutionary patterns can also lead to differences in the number of sites under selection in these genes as well as to differences in the selection regime acting in these (Lopez *et al.*, 2002).

Each species of RABV reservoir can be considered a specific habitat in which each viral lineage adapts and evolves, and the variety of reservoirs currently found for RABV can be considered different adaptive landscapes (Wright 1932) in which different viral populations can adapt in different ways. Sympatric reservoirs for different species can be considered isolated environments that differ from each other in terms of the viruses for which they are reservoirs even though they occupy the same habitat (Wasik and Turner, 2013).

The separation of RABV into bat-related and dog-related inferred to have occurred around 200 AD according to the results presented here helped shape specific genetic and phenotypic characteristics and patterns for the viruses in each of these cycles. Nevertheless, this is not taken into account in classifications and descriptions of the epidemiologic cycles of rabies, which are today divided into an urban and a sylvatic cycle (Acha and Szyfres, 2003). Instead, only factors related to reservoirs and the environment are considered, while factors inherent to the etiologic agent are overlooked.
